# Assessment of the Diversity of Medico-Magic Knowledge on Four Herbaceous Species in Benin

**DOI:** 10.1155/2021/6650704

**Published:** 2021-05-31

**Authors:** Hubert Olivier Dossou-Yovo, Valentin Kindomihou, Fifanou Gbèlidji Vodouhè, Brice Sinsin

**Affiliations:** ^1^Laboratory of Applied Ecology, Faculty of Agronomic Sciences, University of Abomey-Calavi, Benin; ^2^Laboratory of Economic and Social Dynamics Analysis (LARDES), Faculty of Agronomy, University of Parakou, BP 123 Parakou, Benin

## Abstract

**Background:**

Ethnobotanical knowledge on four herbaceous species, *Acmella uliginosa* (Sw.) Cass., *Momordica charantia* L., *Phyllanthus amarus* Schumach. & Thonn., and *Scoparia dulcis* L., in Benin was investigated.

**Methods:**

Herbal medicine traders in six different markets were interviewed using a semi-structured questionnaire. The linear regression test was performed to check for the influence of respondent's age on ethnobotanical uses they hold. Relative frequency citation, fidelity level, use value, and Rahman similarity index were calculated to assess the diversity of medico-magic knowledge. The Informant Consensus Factor is not applicable in this study since we are dealing neither with the diversity of medicinal plants used by a community of people nor with a great number of plant species used for medicinal purposes, nor the diversity of plant species used in the treatment of a specific or group of ailments.

**Results:**

The respondent's age did not influence the ethnobotanical uses they hold on the species. All thirty-six informants surveyed traded *Phyllanthus amarus* Schumach. & Thonn., *Momordica charantia* L., and *Scoparia dulcis* L., and the majority traded *Acmella uliginosa* (Sw.) Cass. The respondent's age does not influence the diversity of ethnobotanical uses they hold on the study species. Purchase in traders' own markets was the predominant source of *Phyllanthus amarus* Schumach. & Thonn., *Momordica charantia* L., and *Scoparia dulcis* L. while *Acmella uliginosa* (Sw.) Cass. was mostly purchased in other more distant markets. A noticeable proportion of traders also collect *Phyllanthus amarus* Schumach. & Thonn. and *Momordica charantia* L. from wild populations. *Phyllanthus amarus* Schumach. & Thonn. was the species most demanded by customers followed by *Momordica charantia* L. Traders confirmed the scarcity of all species in recent years and climate change and destruction of natural habitats for logging were the most cited causes. The entire plant of *Phyllanthus amarus* Schumach. & Thonn. was used mainly to treat malaria, diabetes, and constipation, and decoction with oral administration was the most frequent preparation for malaria treatment. To treat diabetes, informants mixed *Phyllanthus amarus* Schumach. & Thonn. with *Momordica charantia* L. used as a decoction with oral administration. *Momordica charantia* L. was also used to treat measles and chicken pox. *Acmella uliginosa* (Sw.) Cass. and *Scoparia dulcis* L. were mostly used for their spiritual use for luck, predominantly by chewing fresh leaves or flowers, and by bathing with the ground plant mixed with soap, respectively. Overall, *Momordica charantia* L. had the greatest use value followed by *Phyllanthus amarus* Schumach. & Thonn. The majority of traders do not plant the species.

**Conclusions:**

The harvesting and trade of the species threaten their natural populations and urgent tools, including *in situ* and *ex situ* conservation, are needed to ensure their long-term sustainable exploitation.

## 1. Background

The green medicine is obviously of major importance to mankind [1–3]. Gathering of high value products including medicinal plants from the wild continues in developing countries [3, 4]. Moreover, medicinal plants harvested from the wild serve as raw materials for commercial pharmaceutical factories [3, 5]. Thus, medicinal plants are important in both developed and developing countries and there is an increasing need to gather knowledge related to their exploitation and to define conservation strategies for long-term exploitation. Amongst medicinal plants, herbs have a great importance [6, 7] as they are accessible to many people.

Ethnobotanical indices are worldwide applied to evaluate ethnobotanical knowledge on plant species [8–10]. Indeed, quantitative ethnobotany is concerned with measuring the importance of plants and vegetation for human well-being, and it relies on cultural significance indices as quantitative tools for qualitative data [11–13]. Because this approach is likely to generate data that lend themselves to hypothesis testing, statistical validation, and comparative analysis [10], applying specific ethnobotanical indices may serve in analysing the range of information related to plants used for medico-magic purposes. The present research focused on four herbaceous species highly used as medicine in Benin but still less documented, *Acmella uliginosa* (Sw.) Cass., *Momordica charantia* L., *Phyllanthus amarus* Schumach. & Thonn., and *Scoparia dulcis* L. These species have been reported as used in green medicine in many parts of the world [14–17]. Fanou et al. [18] have recently recorded *Momordica charantia* L. among plants used in the treatment of candidiasis in Southern Benin. *Phyllanthus amarus* Schumach. & Thonn. is used by pregnant women in Southern Benin to treat diabetes [19]. Boko-Haya et al. [20] reported the ethnic differences in use of *Phyllanthus amarus* Schumach. & Thonn. in Northern Benin and many years ago, this species was also tested for its antiplasmodial activity in Benin [21]. So there is a need to further documenting the ethnobotanical information on the research species. The aim of the present research was to investigate the medico-magic knowledge held by herbal medicine traders on these herbaceous species in Southern Benin through the application of four ethnobotanical indices, i.e., relative frequency citation [22, 23], fidelity level [24–26], use value [22, 23, 27], and the Rahman similarity index [28].

## 2. Methods

### 2.1. Study Species

Samples of the four research species were certified at the National Herbarium of Benin at the University of Abomey-Calavi and a voucher number was obtained for each species [*Acmella uliginosa* (Sw.) Cass., voucher number YH 532/HNB; *Momordica charantia* L., voucher number YH 530/HNB; *Phyllanthus amarus* Schumach. & Thonn., voucher number YH 529/HNB; *Scoparia dulcis* L., voucher number YH 531/HNB]. Samples of the four study species observed by the author for confirmation in the framework of this research were those in the possession of the herbal medicine traders. During the data collection, the vernacular name of each species in Fon was used [29]. The corresponding author took and retained photographs of the research species during interviews.

*Phyllanthus amarus* Schumach. & Thonn. (Hinlinwé in Fon) belongs to Euphorbiaceae, is an annual herb, 60–75 cm tall, and quite glabrous. Its roots are stout and woody; the stems are branched at the base and angular with numerous leaves. The plant naturally grows in tropical and subtropical climates on well drained sandy-loam soils [30]. It protects the liver and limits the effects of viral hepatitis A, B, and C, reduces type 2 diabetes, helps fighting cancer, and detoxifies the body [31].

*Acmella uliginosa* (Sw.) Cass. (Awélékpékpé in Fon), as an Asteraceae, is an annual broadleaf herb. It grows up to 0.2–1 m, and the flowers are co-linear, with opposite leaves, lanceolate, narrowly ovate or ovate, 1–8.5 cm long, 0.3–3 cm wide, base attenuate, margins sinuate to dentate, apex acute to acuminate, glabrous to sparsely pilose, leaf petiole 0.2–3.2 cm long, narrowly winged. It is a fast-growing herb and under favourable conditions plants may grow copiously [32, 33].

*Momordica charantia* L. (Yinsinkin in Fon), from Cucurbitaceae, commonly called bitter-melon or ampalaya, grows 12 to 20 cm long. Its fruits ripen from green to yellow and ripe fruits are ornamentally attractive but malodorous [34]. It is a pantropical vegetable originated in tropical Africa [35]. Fruit also is carminative, tonic, stomachic, aphrodisiac, anthelmintic, astringent to bowels and useful in treatment of syphilis, rheumatism, spleen troubles, and effectiveness in treating diabetes [36].

*Scoparia dulcis* L. (Viviman téton in Fon) is a Scrophulariaceae occurring widely in Africa. It is a terrestrial, annual, erect herb, up to 75 cm tall. Roots are white or brown, fibrous. Stems are erect, quadrangular, grooved, solid, and glabrous. Stipules are absent. Leaves are simple, not lobed or divided, opposite or whorled, stalked, lanceolate to obovate, both surfaces glabrous, with dark dots underneath, margin coarsely dentate, apex acute, base acute to attenuate. Flowers are bisexual, solitary or with few together, axillary, petals 4, white. The stem is polygonal and full. It is often woody at the base. It is hairless. The leaves are opposite or verticillate in three. They are simple and sessile. The lamina is oblanceolate, 2.5 to 5 cm long and 1.5 cm wide [37–39]. It is considered to be one of such plants which have remarkable curative property [40].

### 2.2. Ethnobotanical Data Collection

Surveys were conducted in some of the most populated towns of Southern Benin, and with herbal medicine traders in some of the most popular herbal medicine markets. These were the Pahou and Zobê markets in Ouidah District, with 445 inhabitants/sq km (the Atlantic Department), the Cococodji and Godomey markets in the Abomey-Calavi District, 1,010 inhabitants/sq km (the Atlantic Department), and Vêdoko and Dantokpa markets in Cotonou district with 8,595 inhabitants/sq km (Department of Littoral). The aim of our research was explained to the responsible of each market in order to obtain the approval to conduct surveys. Similarly, the aim of the study was explained to each trader to get the verbal consent to participate in the research. Six herbal medicine traders were randomly selected in each market and ethnobotanical data were gathered from them on each of the four study species. A semi-structured questionnaire was developed specifically for this study and was used to collect data on the trade, sources, and importance of each species. Similar to the ranking in terms of demand, in recent time, of woody species by wood carvers regardless the type of arts they serve for [41], herbal medicine traders were asked to rank the four species according to consumer demand regardless the ailments treated with. This ranking aimed at assessing the importance of each species to the local populations and would help assume the likely pressure towards species in their natural ecosystems. Traders were also questioned about the availability versus scarcity of each species in recent years. The various causes of scarcity were also recorded. In addition, herbal medicine traders were asked to list all diseases, disorders, and magic issues treated using each species as well as all types of preparation used for these purposes. In order to assess traders' contribution to the research species conservation, the plantation or *in situ* conservation of each species by traders and their motives were investigated. Each time an informant confirmed the trade of a species, they were asked to show a sample for positive identification. All interviews were conducted in the local language, Fon, well-spoken and understood by all informants. Verbal approval to undertake the survey was obtained from each market administration, and informed consent was obtained from each participating trader.

### 2.3. Statistical Analysis of the Influence of the Respondent's Age on the Diversity of Ethnobotanical Uses

In order to test the influence of the respondent's age on the diversity of uses that they hold on the study species, a linear regression analysis was performed using R 4.0.2 package at 0.05 level of probability. The age of each respondent was considered as well as the number of use reports per species and the total number of uses mentioned by each respondent.

### 2.4. Ethnobotanical Indices Calculation for Data Analysis

(a) The relative frequency citation (RFC) [22, 23] was calculated using the following formula:
  (1) $$\begin{array}{c} \text{ RFC}=\frac{n_{i}}{N}\;\times 100, \end{array}$$
where *n*_*i*_ is the number of informants who mentioned the concerned species, thing, or aspect and *N* is the total number of surveyed traders.
(b) The fidelity level (FL) [24–26] was determined for the diseases as follows:
  (2) $$\begin{array}{c} \text{FL}\left(\%\right)=\frac{I_{P}}{I_{U}}\times 100, \end{array}$$
where *I*_*P*_ is the number of informants who mentioned the use of a species for a specific disease, disorder, or magic issue and *I*_*U*_ is the total number of informants who mentioned the species for any use. The FL was also calculated in a similar way for the types of preparation recorded for each disease, disorder, or magic issue. The FL served to assess the informants' preference for a species to treat a specific disease, disorder, or magic issue. It also served to assess the preference for a specific type of preparation. FL ranges from zero to a hundred percent and a value close to 100% means a high preference.
(c) The use value (UV) of each species was determined [22, 23, 27].
  (3) $$\begin{array}{c} \text{UV}=\frac{n_{d}}{I_{U}}, \end{array}$$
where *n*_*d*_ is the total number of use reports (diseases, disorders, or magic utilization) mentioned for a species and *I*_*U*_ is the total number of informants who mentioned the species for any use. The use value served to compare the relative importance of the study species in terms of uses.
(d) The ethnobiological Rahman similarity index (RSI) [28], to assess the similarity between species in terms of uses, was calculated as follows:
  (4) $$\begin{array}{c} \text{RSI }\left(\%\right)=\frac{n_{c}}{n_{a}+n_{b}-n_{c}}\times 100. \end{array}$$
This formula is similar to the Jaccard Similarity Index. Rahman et al. [28] considered an ailment recorded in two communities and treated with a number of medicinal plant species, species commonly used in both communities. In the present research, this approach was adapted to similarity of uses of pairs of species with, respectively, *n*_a_ and *n*_b_ as number of use reports in species a and b and, the number of common uses recorded for the two species as *n*_c_. RSI can range from 0 to 100% and an RSI lower than 50% means low similarity in terms of uses between the two species while an RSI higher than 50% indicates a high similarity of uses between the two species.

## 3. Results

### 3.1. Influence of Respondent's Age on Ethnobotanical Uses

A total of 36 traders were surveyed and the linear regression test revealed an absence of influence of the respondent's age on the diversity of ethnobotanical uses they hold on the study species. In fact, there were age and uses of *Phyllanthus amarus* Schumach. & Thonn. (*F*-statistic = 1.28; DF = 34 and *P*=0.26), age and uses of *Acmella uliginosa* (Sw.) Cass. (*F*-statistic = 0.04; DF = 34 and *P*=0.82), age and uses of *Momordica charantia* L. (*F*-statistic = 1.88; DF = 34 and *P*=0.17), age and uses of *Scoparia dulcis* L. (*F*-statistic = 2.34; DF = 34 and *P*=0.13), and age and the sum of uses of all species (*F*-statistic = 0.23; DF = 34 and *P*=0.63).

### 3.2. Sources of the Species Traded

The 36 informants traded *Phyllanthus amarus* Schumach. & Thonn., *Momordica charantia* L., and *Scoparia dulcis* L. while 95% of informants traded *Acmella uliginosa* (Sw.) Cass. Samples of each species were shown by each trader as proof. With regard to the sources of each species traded (Table 1), six different sources were recorded and the majority of informants purchase *Phyllanthus amarus* Schumach. & Thonn., *Acmella uliginosa* (Sw.) Cass., and *Momordica charantia* L. in both their own markets and other distant markets. In addition to these sources, a relatively large proportion of traders collect plants from wild populations of *Phyllanthus amarus* Schumach. & Thonn. and *Momordica charantia *L. Traders mostly get *Scoparia dulcis* L. by purchasing it in their own markets and collecting the species from the wild. The single most important source of each species reported by herbal medicine traders was their own market for *Phyllanthus amarus* Schumach. & Thonn. (44% of traders), *Momordica charantia* L. (50% of traders), and *Scoparia dulcis* L. (39% of traders), while for *Acmella uliginosa* (Sw.) Cass., it was distant markets (39% of traders).

### 3.3. Ranking of the Species in terms of Demand and Availability

**Figure 1:**
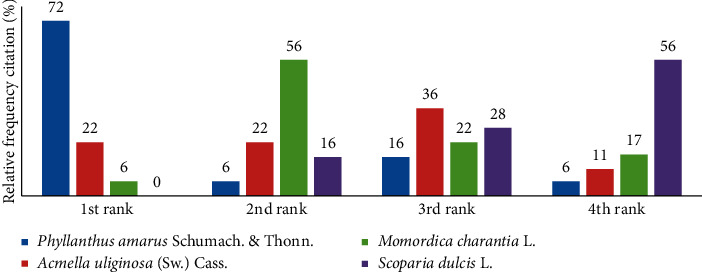
Ranking of the study species by demand according to their traders (number of traders = 36 except for *Acmella uliginosa* (Sw.) Cass., where *n* = 34).

With regard to their demand, *Phyllanthus amarus* Schumach. & Thonn. was ranked as first by 72% of traders, *Acmella uliginosa* (Sw.) Cass. by 22%, *Momordica charantia* L. by 6%, and *Scoparia dulcis* L. by 0% (Figure 1). The majority of surveyed traders confirmed the scarcity rather than easy availability of all four species in recent years. The percentage of traders reporting scarcity was 94% for *Momordica charantia *L., 89% for *Phyllanthus amarus* Schumach. & Thonn., 83% for *Scoparia dulcis* L. and 67% for *Acmella uliginosa* (Sw.) Cass. Eight different factors causing scarcity were reported by the herbal medicine traders (Table 2). Climate change and destruction for logging were the top two causes for all four species with overexploitation also mentioned as an equal second cause for *Acmella uliginosa* (Sw.) Cass.

### 3.4. Uses Recorded for Each Species

Ten uses were recorded for *Phyllanthus amarus* Schumach. & Thonn. parts or the entire plant (Table 3). The most frequently (FL in %) reported use was to treat malaria using the entire plant as medicine, followed by treatment of diabetes, while the same proportion of traders reported the use of this species to treat constipation and stomach aches. Most informants (14 out 16; 88%) who mentioned use of *Phyllanthus amarus* Schumach. & Thonn. to treat diabetes insisted on its combination with *Momordica charantia *L. Three spiritual uses and one medicinal use were recorded for *Acmella uliginosa* (Sw.) Cass. (Table 4). All herbal medicine traders reported the spiritual utilization of this species for luck, but it was also used to treat mouth sores. Fifteen uses were recorded for *Momordica charantia* L. (Table 4). All traders mentioned the use of this species to treat measles and chicken pox. All informants who mentioned the use of this species to treat diabetes (39%) insisted on its mixture with *Phyllanthus amarus* Schumach. & Thonn. *Scoparia dulcis* L. was recorded as used to treat eight diseases, disorders, and magic issues (Table 4). The most reported use of this species (FL = 86%) was its spiritual use for luck.

### 3.5. Relative Importance of Species and Their Similarity in terms of Uses

All species exhibited a use value lower than 0.5 (Table 3). However, *Momordica charantia* L. was relatively the most important in terms of uses. The Rahman similarity index for uses of pairs of species (Table 3) confirmed an absence of similarity of use between *Phyllanthus amarus* Schumach. & Thonn. and both *Scoparia dulcis* L. and *Acmella uliginosa* (Sw.) Cass., and also between *Momordica charantia* L. and *Acmella uliginosa* (Sw.) Cass. However, there was a moderate similarity of use between *Phyllanthus amarus* Schumach. & Thonn. and *Momordica charantia* L. with both used to treat stomach complaints, malaria, diabetes, and constipation. There were very low similarities of use between *Momordica charantia* L. and *Scoparia dulcis* L., and between *Acmella uliginosa* (Sw.) Cass. and *Scoparia dulcis* L. with these pairs of species used in common to treat malaria and for good luck, respectively.

### 3.6. Preparation and Modes of Administration

#### *3.6.1. Phyllanthus amarus* Schumach. & Thonn

Based on the Fidelity Level, the three major conditions treated with this species were malaria, diabetes, and constipation, the last chosen in this table instead of stomachache although having equal FL (Table 5). Four types of preparation were mentioned by informants for malaria treatment and three for the other two conditions. For each condition, the majority of traders suggested the use of the entire plant as a decoction and the route of administration was oral. An infusion of the entire plant in a traditional alcoholic drink or in hot water was also mentioned by a minority of the traders to treat all three conditions. A mixture of the dried, powdered plant in an alcoholic drink was reported as a treatment only for malaria.

#### *3.6.2. Acmella uliginosa* (Sw.) Cass

Only the spiritual use of this species for luck was considered. Seven preparations were mentioned by medicinal plant traders (Table 5). The most commonly reported was the chewing of the leaves followed by the chewing of flowers early in the morning to request for good luck in any kind of business.

#### *3.6.3. Momordica charantia *L

The three major conditions treated using this species were measles, chicken pox, and diabetes (Table 5). Informants insisted that the first two diseases shared the same preparations. Seven modes of preparation were recorded for each of these diseases and the most common was grinding the plant (leaves and stems) in the traditional alcoholic drink, “sodabi.” Regarding diabetes, similarly to the findings for *Phyllanthus amarus* Schumach. & Thonn. with which this species was usually combined, three types of preparation were noted, the most common being the decoction of the plant (leaves and stems) with oral administration.

#### *3.6.4. Scoparia dulcis* L

Only the magic use of this species for luck was considered and six preparations were reported (Table 5). Based on the Fidelity Level, the most common preparation, mentioned by a majority of the traders, involved grinding the plant (leaves and stems) and mixing it with soap for bathing. So, the route of administration was herbal bathing. This was said by the traders to bring good luck to business activities.

### 3.7. Traders' Contribution to Species Conservation

**Figure 2:**
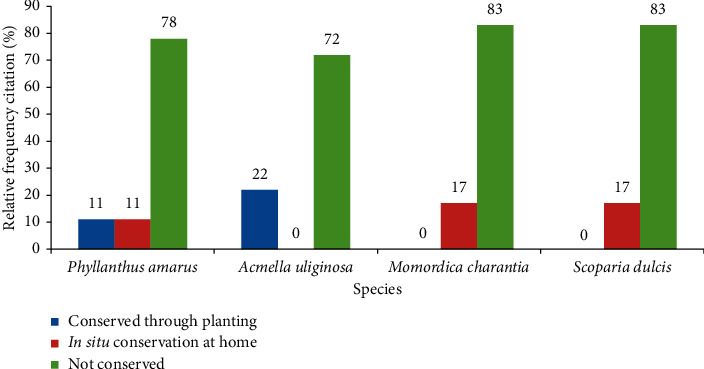
Informants' contribution to species conservation (number of traders = 36 except for *Acmella uliginosa* (Sw.) Cass., where *n* = 34).

The majority of the traders did not plant the four research species (Figure 2). However, a low proportion of traders confirmed that they planted *Acmella uliginosa* (Sw.) Cass. and *Phyllanthus amarus* Schumach. & Thonn. for trade and personal use. *In situ* conservation of the naturally grown populations of all species at home except *Acmella uliginosa* (Sw.) Cass. was reported by a small proportion of traders.

## 4. Discussion

### 4.1. Influence of Respondent's Age on Ethnobotanical Uses

Our study revealed an absence of the age influence on the ethnobotanical uses hold by herbal medicine traders on the study species. However, with regard to the size of our sample (36), authors assume that another study undertaken on the same species with a greater sample of respondents can reveal other trends. Further investigations on our research species throughout Benin are needed to state a big range of ethnobotanical knowledge.

### 4.2. Sources of Plant Species Traded

All informants confirmed the trade of *Phyllanthus amarus* Schumach. & Thonn., *Momordica charantia* L., and *Scoparia dulcis* L. while 94% traded *Acmella uliginosa* (Sw.) Cass. These findings confirmed the medico-magic importance of these four herbaceous species to indigenous people in Benin. In addition, the trade of these species may generate a considerable income to traders. There is an increasing demand for plants for herbal drugs, natural health products, and secondary metabolites throughout the world [42]. The majority of traders purchased the four species in their own markets, meaning a close relationship and collaboration between medicinal plant harvesters and local traders. Elsewhere, the relationship between wholesalers and traders of medicinal plants in South Asia seemed to be exploitative [43]. There is a need to better understand this relationship in Africa, especially in Benin. In all cases, there were fewer traders reporting purchasing the four medicinal plants in distant markets. The collection from wild populations of three of the four species, *Phyllanthus amarus* Schumach. & Thonn., *Momordica charantia* L., and *Scoparia dulcis* L., confirmed the connection of local traders with their environment. Moreover, since there is no control of herbaceous medicinal plant collection from the wild, this harvesting may threaten the conservation of the concerned species. Consequently, further investigations are needed on the harvesting and trade of herbaceous medicinal plants to ensure their long-term sustainable conservation.

### 4.3. Ranking of the Four Species in terms of Demand and Availability

The ranking of *Phyllanthus amarus* Schumach. & Thonn. as most demanded by the greatest number of traders showed the importance of this species in health care of the local population. Similarly, *Momordica charantia* L. also has a high importance. The high demand for these two species suggests that higher pressure is likely on the conservation of these species than on the other two. The use of species belonging to the genus *Phyllanthus* as green medicine is becoming more popular [15]. *Momordica charantia* L. is widely used and in demand all over the world for its medicinal potency [44] resulting in over exploitation of its wild populations. Similar to our findings related to *Acmella uliginosa* (Sw.) Cass., other Asteraceae species belonging to the genus *Spilanthes* are widely used in traditional medicine in various cultures [45]. Knowledge related to the impact of climate change on plant species availability [46] was confirmed by local traders who perceived climate change to be a contributing factor to species scarcity. Additionally, the destruction of species habitats for logging reported by traders confirmed threats to forest biodiversity due to both habitat loss and degradation of forest ecosystems [47, 48]. Not all medicinal plant species are affected in the same way by harvesting pressure so research is needed to assess the factors affecting the scarcity of each species studied. Herbal traders who reported the planting of *Acmella uliginosa* (Sw.) Cass. in other gardens confirmed the *ex situ* conservation of this species. As a result, further investigations should be conducted in gardens to assess the ecological patterns and horticultural practices of these plantings. This will serve to promote cultivation of the species planting throughout the country in gardens.

### 4.4. Diversity of Diseases, Disorders, and Magic Utilization of Species

The large number of uses recorded for *Phyllanthus amarus* Schumach. & Thonn. and *Momordica charantia* L. revealed that herbal traders hold a diversity of knowledge on these species. The medicinal potency of *Momordica charantia* L. has been widely reported [44, 49, 50] including its use to treat diabetes as reported in the present study. The use of the species to treat measles was also reported in Nigeria [16], as well as its action against inflammatory diseases [17]. The combination of *Phyllanthus amarus* Schumach. & Thonn. with *Momordica charantia* L. for the treatment of diabetes corroborates findings of Sarin et al. [15] who mentioned diabetes among ailments treated with *Phyllanthus* species. As recorded in the present research, indigenous people in Nigeria used fresh leaves of *Momordica charantia* L. in various preparations to treat measles [16]. The medicinal use of this species seems common along the West African coast. Since diabetes is mentioned among the five most lethal diseases in the world [51], further research is needed on the pharmacognosy of *Phyllanthus amarus* Schumach. & Thonn., *Momordica charantia* L. and their combination in treating diabetes. Similar to the present results, the use of *Phyllanthus* species in the treatment of malaria, liver disorders, and constipation was also stated by Sarin et al. [15]. Malaria is one of the most lethal diseases in tropical countries so the long-term conservation of medicinal plants, especially *Phyllanthus amarus* Schumach. & Thonn. active in treating malaria in Benin, provides a guarantee for the treatment of this dreadful disease.

The magic utilization recorded for *Acmella uliginosa* (Sw.) Cass. in this research was a proof of traditional belief that African populations assign to plants. *Scoparia dulcis* L., similar to *Acmella uliginosa* (Sw.) Cass., was known as magically efficient by herbal traders. This confirmed the connection of Africans with their nature and the conservation of traditional beliefs across generations. In fact, similar to our findings, Mafimisebi and Oguntade [52] reported magico-religious practices using plant species in Nigeria. Recently, Dossou-Yovo et al. [3] reported the magico-religious practices and rituals using mangrove plant species in Benin. Since people believe that those species bring good luck, this is a strong argument to be used to promote their cultivation and conservation throughout the country. Elsewhere, *Scoparia dulcis* L. has recently been reported in the treatment of jaundice, stomach problems, and diabetes [40].

### 4.5. Relative Importance of Species and Their Similarity in terms of Uses

Despite being the most frequently demanded, *Phyllanthus amarus* Schumach. & Thonn. did not have the highest use value. In contrast, *Momordica charantia* L. ranked second for demand had a higher use value than *Phyllanthus amarus* Schumach. & Thonn. The relative importance and high demand confirmed the need for sustainable conservation strategies for *Phyllanthus amarus* Schumach. & Thonn. and *Momordica charantia* L. as medicinal plants. *Acmella uliginosa* (Sw.) Cass. and *Scoparia dulcis* L. were the least important in terms of uses. Although there was a low similarity in terms of use between *Phyllanthus amarus* Schumach. & Thonn. and *Momordica charantia* L., the RSI value indicated that 19% ailments and disorders could be treated using either *Phyllanthus amarus* Schumach. & Thonn. or *Momordica charantia* L. *Acmella uliginosa* (Sw.) Cass. and *Scoparia dulcis* L. with an RSI value of 9% also had a low similarity of use. However, the possible substitution of one medicinal plant for another justifies further study as it might contribute to conservation by relieving pressure of exploitation on wild populations of scarce and most threatened medicinal plant species in tropical countries.

### 4.6. Preparation and Routes of Administration

Similar to the present findings, aqueous extracts of *Phyllanthus amarus* Schumach. & Thonn. were reported as more efficient than ethanol extracts for the treatment of malaria by Ajala et al. [14]. The preference of traders for decoctions with an oral route of administration against malaria matches western medical knowledge and confirmed that indigenous knowledge on green medicine has a great role to play in fighting dangerous diseases worldwide. *Phyllanthus amarus* Schumach. & Thonn. was also recorded as the most commonly used in diabetes treatment, predominantly as a decoction with oral route of administration by Thai communities [51]. Constipation is a stomach disorder very often related to dysfunctioning of the liver. Given that excessive alcohol consumption is damaging to the liver [53], the authors question the wisdom of recommending extracts of medicinal plants in alcohol for the treatment of this condition. *Acmella uliginosa* (Sw.) Cass. and *Scoparia dulcis* L. were recorded mainly for their spiritual use for luck and the chewing of leaves or flowers, and the mixture of the ground plant with soap for bathing were the predominant methods of use. Elsewhere, Asteraceae species of the genus *Spilanthes* were reported as used for more than 60 disorders including parasitic diseases [45]. Herbal bathing as route of administration recorded in this research was also reported by Sabran et al. [54]. The harvesting of fresh flowers of *Acmella uliginosa* (Sw.) Cass. for chewing compromises the reproductive capacity of the species since flowers are reproductive organs.

### 4.7. Traders' Contribution to Species Conservation

There is evidence of the important role that *in situ* and *ex situ* conservation plays in the conservation of plant genetic resources [45]. However, the majority of traders do not plant the medicinal plant species that they sell. Therefore, there is an urgent need to promote the species plantation or the *in situ* conservation of naturally grown populations of the study species. Traders should be trained and sensitized on tools for the planting of *Phyllanthus amarus* Schumach. & Thonn. whenever possible in order to reduce pressure on its wild populations. *Momordica charantia* L., exhibiting a relative high importance in this study, should also be conserved by traders wherever it grows. Although few uses were recorded for *Acmella uliginosa* (Sw.) Cass. and *Scoparia dulcis* L., it is recommended that traders also contribute to the *in situ* and *ex situ* conservation of these species. Those traders who confirmed the planting and conservation of naturally grown populations of the research species should be encouraged to contribute to training and dissemination of conservation tools for these species. Herbaceous species, especially those used as medicine in Benin, are often overlooked by conservation programs. The authors strongly suggest that greater attention should be paid to herbaceous medicinal plants in Benin in order to contribute to their conservation.

## 5. Conclusions

The ethnobotanical uses held by the herbal medicine traders were not influenced by their age. The four species were mostly purchased by traders in their own markets. *Phyllanthus amarus* Schumach. & Thonn., *M*omordica *Charantia* L., and *Scoparia dulcis* L. were also collected from the wild by herbal medicine traders for sale. *Phyllanthus amarus* Schumach. & Thonn. was the most demanded followed by *Momordica charantia* L. A diversity of diseases and disorders were traded using the four species, and *Momordica charantia* L. had the highest use value followed by *Phyllanthus amarus* Schumach. & Thonn. There was a very low similarity between the research species in terms of uses. Most of the surveyed traders do not plant the research species so there is an urgent need to promote their planting and sustainable conservation.

## Figures and Tables

**Table 1:** Sources of the study species according to traders.

	Purchase in their markets	Purchase in nearby markets	Purchase in distant markets	Purchase in gardens	Collection from the wild populations	Collection from own gardens
	RFC (%)^*∗*^	RFC (%)	RFC (%)	RFC (%)	RFC (%)	RFC (%)
*Phyllanthus amarus* Schumach. & Thonn.	83	17	50	6	33	0
*Acmella uliginosa* (Sw.) Cass.	72	11	50	17	0	6
*Momordica charantia* L.	78	11	44	0	44	6
*Scoparia dulcis* L.	56	11	33	0	44	0

^*∗*^RFC (%) = relative frequency citation (%). Number of traders = 36.

**Table 2:** Causes of scarcity of the study species according to traders.

	RFC (%)^*∗*^							
	Destruction for logging	Climate change	Population growth	Overexploitation	International trade	Overexploitation due to COVID-19 pandemic	Cultivation of humid areas	Loss of soil fertility
*Phyllanthus amarus* Schumach. & Thonn.	44	67	6	17	6	11	0	6
*Acmella uliginosa* (Sw.) Cass.	17	44	0	17	0	0	0	0
*Momordica charantia* L.	39	78	6	17	0	11	0	0
*Scoparia dulcis* L.	28	67	6	6	0	0	6	6

^*∗*^RFC (%) = relative frequency citation (%). Number of traders = 36 except for *S. uliginosa* where *n* = 34.

**Table 3:** Relative importance of the study species (use value).

Species	Total no. of uses	Use value (UV)	Number of shared uses/Rahman similarity index (RSI %)		
			*Phyllanthus amarus* Schumach. & Thonn.	*Acmella uliginosa* (Sw.) Cass.	*Momordica charantia* L.
*Phyllanthus amarus* Schumach. & Thonn.	10	0.27	—	—	—
*Acmella uliginosa* (Sw.) Cass.	4	0.11	00	—	—
*Momordica charantia* L.	15	0.41	4	0	—
			19	0	
*Scoparia dulcis* L.	8	0.22	0	1	1
			0	5	5

Number of traders = 36 except for *S. uliginosa* where *n* = 34.

**Table 4:** Fidelity level (FL%) of recorded diseases, disorders, and magic uses per study species.

Situations types	Plant species and FL (%)^*∗*^				
	List of social situations recorded	*Phyllanthus amarus* Schumach. & Thonn.	*Acmella uliginosa* (Sw.) Cass.	*Momordica charantia* L.	*Scoparia dulcis* L.
Diseases	Diabetes	44	0	39	0
	Infant intelligence stimulation	0	0	0	6
	Malaria	67	0	22	9
	Tiredness of old persons	6	0	0	0

Magic utilization	Spiritual use for luck	0	100	0	86
	Spiritual use to in Vodun^†^	0	12	22	0
	Spiritual use for self-protection	0	6	0	0
	Spiritual use for love	0	0	0	3

Infections	Chicken pox	0	0	100	0
	Constipation	33	0	14	0
	Cough	0	0	0	17
	Deworming	6	0	0	0
	Dog bite	0	0	6	0
	Eye infection	0	0	6	0
	Fever	0	0	14	0
	Mouth sore	0	6	0	0
	Measles	0	0	100	0
	Laxative use	0	0	8	0
	Skin infection	0	0	6	0
	Stomach infection	28	0	17	0
	Vaginal infection	0	0	6	0
	Urinary tract infection	0	0	6	0

Disorders	Male aphrodisiac	0	0	0	11
	Female aphrodisiac	0	0	0	11
	Male impotence	0	0	0	6
	Stomachache	33	0	0	0
	Liver disorder	6	0	0	0

^†^A West African religion. ^*∗*^FL (%) = fidelity level (%). Number of traders = 36 except for *S. uliginosa* where *n* = 34.

**Table 5:** Fidelity level (FL %) of the preparations used for the main purposes for each species.

Preparation	FL (%)^*∗*^							
	*Phyllanthus amarus* Schumach. & Thonn.		*Acmella uliginosa* (Sw.) Cass.		*Momordica Charantia* L.		*Scoparia dulcis* L.	
	Malaria *n* = 24^†^	Diabetes *n* = 16	Constipation *n* = 12	Luck *n* = 34	Measles *n* = 36	Chicken pox *n* = 36	Diabetes *n* = 14	Luck *n* = 31
Decoction of the entire plant for drinking	88	88	50	—	—	—	—	—
Entire plant dried, powdered, and mixed in traditional alcoholic drink “sodabi”	17	0	0	—	—	—	—	—
Entire plant infused in traditional alcoholic drink	21	13	37	—	17	17	14	—
Entire plant infused in hot water for drinking	8	25	17	—	0	0	29	—
Leaves chewed	—	—	—	82	—	—	—	16
Flowers chewed	—	—	—	62	—	—	—	—
Leaves and flowers chewed	—	—	—	6	—	—	—	—
Entire plant infusion in traditional alcoholic drink	—	—	—	24	—	—	—	—
Entire plant pounded and mixed with soap for bathing	—	—	—	24	—	—	—	13
Infusion of leaves and stems in water used as a spray	—	—	—	6	—	—	—	19
Infusion of pounded entire plant in water used as a spray	—	—	—	24	—	—	—	6
Decoction of leaves and stems in water for drinking	—	—	—	—	25	25	86	—
Leaves and stems ground in water for bathing	—	—	—	—	17	17	0	—
Plant ground in water for drinking	—	—	—	—	22	22	0	—
Plant ground in water for drinking and bathing	—	—	—	—	28	28	0	—
Leaves and stems ground in traditional alcoholic drink “sodabi”	—	—	—	—	47	47	0	—
Leaves and stems ground in natural lemon juice for drinking	—	—	—	—	11	11	0	
Entire plant ground in water for bathing	—	—	—	—				10
Entire plant ground and mixed with soap for bathing	—	—	—	—				58

^*∗*^FL (%) = fidelity level (%). ^†^Number of traders mentioning this use. Total number of traders = 36 except for *S. uliginosa* where *n* = 34.

## Data Availability

The data used in the study are available from the corresponding author upon request.

## Abbreviations

FL: Fidelity level
IUCN: International Union for Conservation of Nature
RFC: Relative frequency citation
RSI: Rahman similarity index
UV: Use value.
